# Supplementary material for: Synthesis and Biological Activities of Luminescent 5,6-Membered Bis(Metallacyclic) Platinum(II) Complexes
Source: Molecules. 2023 Aug 31;28(17):6369. doi: 10.3390/molecules28176369 (PMC10489632; doi:10.3390/molecules28176369)

checkCIF/PLATON report

You have not supplied any structure factors. As a result the full set of tests cannot be run.

THIS REPORT IS FOR GUIDANCE ONLY. IF USED AS PART OF A REVIEW PROCEDURE FOR PUBLICATION, IT SHOULD NOT REPLACE THE EXPERTISE OF AN EXPERIENCED CRYSTALLOGRAPHIC REFEREE.

No syntax errors found.   CIF dictionary   Interpreting this report

Datablock: 1

Bond precision:	C-C = 0.0191 A		Wavelength=0.71073
Cell:	a=7.2605(2)	b=14.3218(3)	c=18.0040(3)
	alpha=70.891(2)	beta=86.709(2)	gamma=84.755(2)
Temperature:	100 K		

	Calculated	Reported
Volume	1760.80(7)	1760.80(7)
Space group	P 1	P 1
Hall group	P 1	P 1
Moiety formula	2(C38 H35 N4 Pt), C F3 O3 S [+ solvent]	2(C38 H35 N4 Pt), C F3 O3 S
Sum formula	C77 H70 F3 N8 O3 Pt2 S [+ solvent]	C77 H70 F3 N8 O3 Pt2 S
Mr	1634.63	1634.65
Dx,g cm-3	1.542	1.542
Z	1	1
Mu (mm-1)	4.059	4.059
F000	811.0	811.0
F000'	808.08	
h,k,lmax	8,17,21	8,17,21
Nref	12440[6220]	11281
Tmin,Tmax	0.448,0.522	0.864,1.000
Tmin'	0.440	

Correction method= # Reported T Limits: Tmin=0.864 Tmax=1.000
AbsCorr = MULTI-SCAN

Data completeness= 1.81/0.91   Theta(max)= 24.998

R(reflections)= 0.0323(10450)   wR2(reflections)= 0.0740(11281)

S = 1.053   Npar= 795

The following ALERTS were generated. Each ALERT has the format
test-name_ALERT_alert-type_alert-level.
Click on the hyperlinks for more details of the test.

Alert level C

PLAT090_ALERT_3_C Poor Data / Parameter Ratio (Zmax > 18) .... 7.82 Note
PLAT342_ALERT_3_C Low Bond Precision on C-C Bonds .... 0.01912 Ang.
PLAT362_ALERT_2_C Short C(sp3)-C(sp2) Bond C4 - C18 . 1.36 Ang.
PLAT363_ALERT_2_C Long C(sp3)-C(sp2) Bond C7 - C61 . 1.65 Ang.
PLAT369_ALERT_2_C Long C(sp2)-C(sp2) Bond C49 - C74 . 1.53 Ang.

Alert level G

PLAT002_ALERT_2_G Number of Distance or Angle Restraints on AtSite 28 Note
PLAT003_ALERT_2_G Number of Uiso or Uij Restrained non-H Atoms ... 92 Report
PLAT033_ALERT_4_G Flack x Value Deviates > 3.0 * sigma from Zero . 0.185 Note
PLAT083_ALERT_2_G SHELXL Second Parameter in WGHT Unusually Large 11.93 Why ?
PLAT111_ALERT_2_G ADDSYM Detects New (Pseudo) Centre of Symmetry . 96 %Fit
PLAT113_ALERT_2_G ADDSYM Suggests Possible Pseudo/New Space Group P-1 Check
Check Model Parameter Symmmetry for Reflection Data Support
PLAT154_ALERT_1_G The s.u.'s on the Cell Angles are Equal ..(Note) 0.002 Degree
PLAT172_ALERT_4_G The CIF-Embedded .res File Contains DFIX Records 24 Report
PLAT173_ALERT_4_G The CIF-Embedded .res File Contains DANG Records 3 Report
PLAT176_ALERT_4_G The CIF-Embedded .res File Contains SADI Records 6 Report
PLAT178_ALERT_4_G The CIF-Embedded .res File Contains SIMU Records 2 Report
PLAT187_ALERT_4_G The CIF-Embedded .res File Contains RIGU Records 1 Report
PLAT188_ALERT_3_G A Non-default SIMU Restraint Value has been used 0.0100 Report
PLAT191_ALERT_3_G A Non-default SADI Restraint Value has been used 0.0400 Report
PLAT191_ALERT_3_G A Non-default SADI Restraint Value has been used 0.0400 Report
PLAT191_ALERT_3_G A Non-default SADI Restraint Value has been used 0.0400 Report
PLAT191_ALERT_3_G A Non-default SADI Restraint Value has been used 0.0400 Report
PLAT343_ALERT_2_G Unusual sp3 Angle Range in Main Residue for C33 Check
PLAT343_ALERT_2_G Unusual Angle Range in Main Residue for C77 Check
PLAT367_ALERT_2_G Long? C(sp?)-C(sp?) Bond C15 - C77 . 1.54 Ang.
PLAT367_ALERT_2_G Long? C(sp?)-C(sp?) Bond C35 - C77 . 1.60 Ang.
PLAT606_ALERT_4_G Solvent Accessible VOID(S) in Structure .... ! Info
PLAT790_ALERT_4_G Centre of Gravity not Within Unit Cell: Resd. # 3 Note
C F3 O3 S
PLAT794_ALERT_5_G Tentative Bond Valency for Pt1 (II) . 2.18 Info
PLAT794_ALERT_5_G Tentative Bond Valency for Pt2 (II) . 2.08 Info
PLAT860_ALERT_3_G Number of Least-Squares Restraints .... 4899 Note
PLAT868_ALERT_4_G ALERTS Due to the Use of _smtbx_masks Suppressed ! Info
PLAT933_ALERT_2_G Number of HKL-OMIT Records in Embedded .res File 8 Note
PLAT941_ALERT_3_G Average HKL Measurement Multiplicity .... 4.0 Low
PLAT967_ALERT_5_G Note: Two-Theta Cutoff Value in Embedded .res .. 50.0 Degree

0 **ALERT level A** = Most likely a serious problem - resolve or explain
0 **ALERT level B** = A potentially serious problem, consider carefully
5 **ALERT level C** = Check. Ensure it is not caused by an omission or oversight

```
30 ALERT level G = General information/check it is not something unexpected

 1 ALERT type 1 CIF construction/syntax error, inconsistent or missing data
13 ALERT type 2 Indicator that the structure model may be wrong or deficient
 9 ALERT type 3 Indicator that the structure quality may be low
 9 ALERT type 4 Improvement, methodology, query or suggestion
 3 ALERT type 5 Informative message, check
```

It is advisable to attempt to resolve as many as possible of the alerts in all categories. Often the minor alerts point to easily fixed oversights, errors and omissions in your CIF or refinement strategy, so attention to these fine details can be worthwhile. In order to resolve some of the more serious problems it may be necessary to carry out additional measurements or structure refinements. However, the purpose of your study may justify the reported deviations and the more serious of these should normally be commented upon in the discussion or experimental section of a paper or in the "special_details" fields of the CIF. checkCIF was carefully designed to identify outliers and unusual parameters, but every test has its limitations and alerts that are not important in a particular case may appear. Conversely, the absence of alerts does not guarantee there are no aspects of the results needing attention. It is up to the individual to critically assess their own results and, if necessary, seek expert advice.

Publication of your CIF in IUCr journals

A basic structural check has been run on your CIF. These basic checks will be run on all CIFs submitted for publication in IUCr journals (*Acta Crystallographica*, *Journal of Applied Crystallography*, *Journal of Synchrotron Radiation*); however, if you intend to submit to *Acta Crystallographica Section C* or *E* or *IUCrData*, you should make sure that full publication checks are run on the final version of your CIF prior to submission.

Publication of your CIF in other journals

Please refer to the *Notes for Authors* of the relevant journal for any special instructions relating to CIF submission.

PLATON version of 10/05/2023; check.def file version of 10/05/2023

Datablock 1 - ellipsoid plot

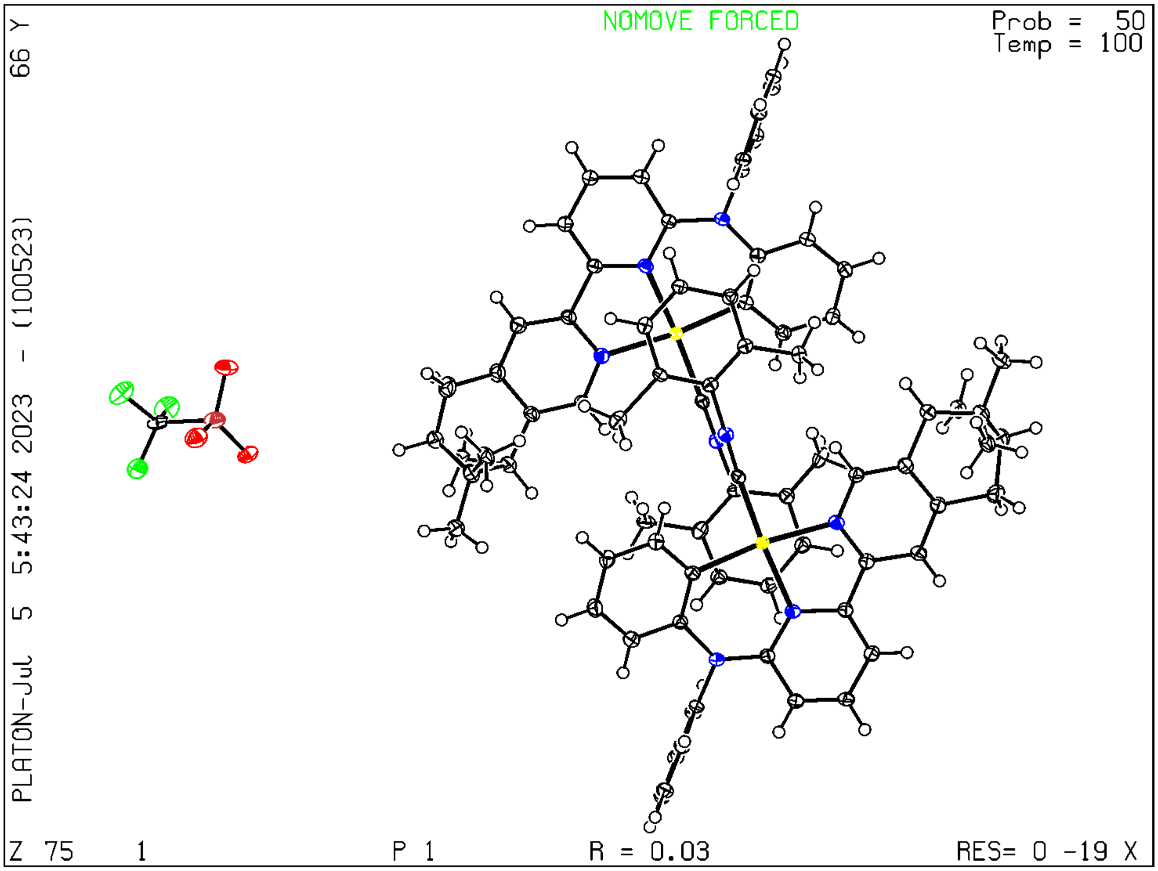

Supplement: Supplementary file 1 [file molecules-28-06369-s001.zip › checkcif-(-)-2.pdf]
