# Supplementary material for: Synthesis and Biological Activities of Luminescent 5,6-Membered Bis(Metallacyclic) Platinum(II) Complexes
Source: Molecules. 2023 Aug 31;28(17):6369. doi: 10.3390/molecules28176369 (PMC10489632; doi:10.3390/molecules28176369)

checkCIF/PLATON report

You have not supplied any structure factors. As a result the full set of tests cannot be run.

THIS REPORT IS FOR GUIDANCE ONLY. IF USED AS PART OF A REVIEW PROCEDURE FOR PUBLICATION, IT SHOULD NOT REPLACE THE EXPERTISE OF AN EXPERIENCED CRYSTALLOGRAPHIC REFEREE.

No syntax errors found. CIF dictionary Interpreting this report

Datablock: 220811lu_lgpz_040300_zcs_0m

```
Bond precision:   C-C = 0.0134 A          Wavelength=0.71073

Cell:             a=11.8935(3)     b=13.8385(4)       c=17.6968(5)
                  alpha=90         beta=91.266(1)     gamma=90
Temperature:      193 K

                 Calculated                   Reported
Volume           2911.97(14)                  2911.97(14)
Space group      P 21                         P2(1)
Hall group       P 2yb                        ?
Moiety formula   C37 H31 N3 Pt                C37 H31 N3 Pt
Sum formula      C37 H31 N3 Pt                C37 H31 N3 Pt
Mr               712.73                       712.74
Dx,g cm-3        1.626                        1.626
Z                4                            4
Mu (mm-1)        4.849                        4.849
F000             1408.0                       1408.0
F000'            1401.79
h,k,lmax         14,16,21                     14,16,21
Nref             10267[ 5367]                 9510
Tmin,Tmax        0.267,0.344                  0.267,0.345
Tmin'            0.225

Correction method= # Reported T Limits: Tmin=0.267 Tmax=0.345
AbsCorr = MULTI-SCAN

Data completeness= 1.77/0.93       Theta(max)= 25.000

R(reflections)= 0.0346( 8293)                     wR2(reflections)=
                                                  0.0970( 9510)
S = 1.045                  Npar= 743
```

The following ALERTS were generated. Each ALERT has the format
test-name_ALERT_alert-type_alert-level.
Click on the hyperlinks for more details of the test.

Alert level C

```
PLAT090_ALERT_3_C Poor Data / Parameter Ratio (Zmax > 18) ........       7.22 Note
PLAT213_ALERT_2_C Atom C10             has ADP max/min Ratio .....        3.9 oblate
PLAT213_ALERT_2_C Atom C13             has ADP max/min Ratio .....        3.6 oblate
PLAT220_ALERT_2_C NonSolvent   Resd 1  C   Ueq(max)/Ueq(min) Range        3.5 Ratio
PLAT220_ALERT_2_C NonSolvent   Resd 2  C   Ueq(max)/Ueq(min) Range        3.9 Ratio
PLAT331_ALERT_2_C Small Aver Phenyl C-C Dist C45      --C50      .       1.37 Ang.
PLAT342_ALERT_3_C Low Bond Precision on  C-C Bonds ..............    0.01342 Ang.
```

Alert level G

```
PLAT003_ALERT_2_G Number of Uiso or Uij Restrained non-H Atoms ...         82 Report
PLAT005_ALERT_5_G No Embedded Refinement Details Found  in the CIF     Please Do !
PLAT033_ALERT_4_G Flack x Value Deviates > 3.0 * sigma from Zero .      0.153 Note
PLAT066_ALERT_1_G Predicted and Reported Tmin&Tmax Range Identical          ? Check
PLAT093_ALERT_1_G No s.u.'s on H-positions, Refinement Reported as      mixed Check
PLAT111_ALERT_2_G ADDSYM Detects New (Pseudo) Centre of Symmetry .         92 %Fit
PLAT112_ALERT_2_G ADDSYM Detects New (Pseudo) Symm. Elem         a         90 %Fit
PLAT113_ALERT_2_G ADDSYM Suggests Possible Pseudo/New  Space Group      P21/c Check
              Check Model Parameter Symmmetry for Reflection Data Support
PLAT371_ALERT_2_G Long   C(sp2)-C(sp1) Bond  C33      - C34      .       1.46 Ang.
PLAT371_ALERT_2_G Long   C(sp2)-C(sp1) Bond  C68      - C69      .       1.43 Ang.
PLAT794_ALERT_5_G Tentative Bond Valency for Pt1       (II)      .       2.13 Info
PLAT794_ALERT_5_G Tentative Bond Valency for Pt2       (II)      .       2.11 Info
PLAT860_ALERT_3_G Number of Least-Squares Restraints ............         799 Note
PLAT899_ALERT_4_G SHELXL-97  is Deprecated and Succeeded by SHELXL     2019/3 Note
```

0 **ALERT level A** = Most likely a serious problem - resolve or explain
0 **ALERT level B** = A potentially serious problem, consider carefully
7 **ALERT level C** = Check. Ensure it is not caused by an omission or oversight
14 **ALERT level G** = General information/check it is not something unexpected

2 ALERT type 1 CIF construction/syntax error, inconsistent or missing data
11 ALERT type 2 Indicator that the structure model may be wrong or deficient
3 ALERT type 3 Indicator that the structure quality may be low
2 ALERT type 4 Improvement, methodology, query or suggestion
3 ALERT type 5 Informative message, check

It is advisable to attempt to resolve as many as possible of the alerts in all categories. Often the minor alerts point to easily fixed oversights, errors and omissions in your CIF or refinement strategy, so attention to these fine details can be worthwhile. In order to resolve some of the more serious problems it may be necessary to carry out additional measurements or structure refinements. However, the purpose of your study may justify the reported deviations and the more serious of these should normally be commented upon in the discussion or experimental section of a paper or in the "special_details" fields of the CIF. checkCIF was carefully designed to identify outliers and unusual parameters, but every test has its limitations and alerts that are not important in a particular case may appear. Conversely, the absence of alerts does not guarantee there are no aspects of the results needing attention. It is up to the individual to critically assess their own results and, if necessary, seek expert advice.

Publication of your CIF in IUCr journals

A basic structural check has been run on your CIF. These basic checks will be run on all CIFs submitted for publication in IUCr journals (*Acta Crystallographica*, *Journal of Applied Crystallography*, *Journal of Synchrotron Radiation*); however, if you intend to submit to *Acta Crystallographica Section C* or *E* or *IUCrData*, you should make sure that full publication checks are run on the final version of your CIF prior to submission.

Publication of your CIF in other journals

Please refer to the *Notes for Authors* of the relevant journal for any special instructions relating to CIF submission.

PLATON version of 10/05/2023; check.def file version of 10/05/2023

Datablock 220811lu_lgpz_040300_zcs_0m - ellipsoid plot

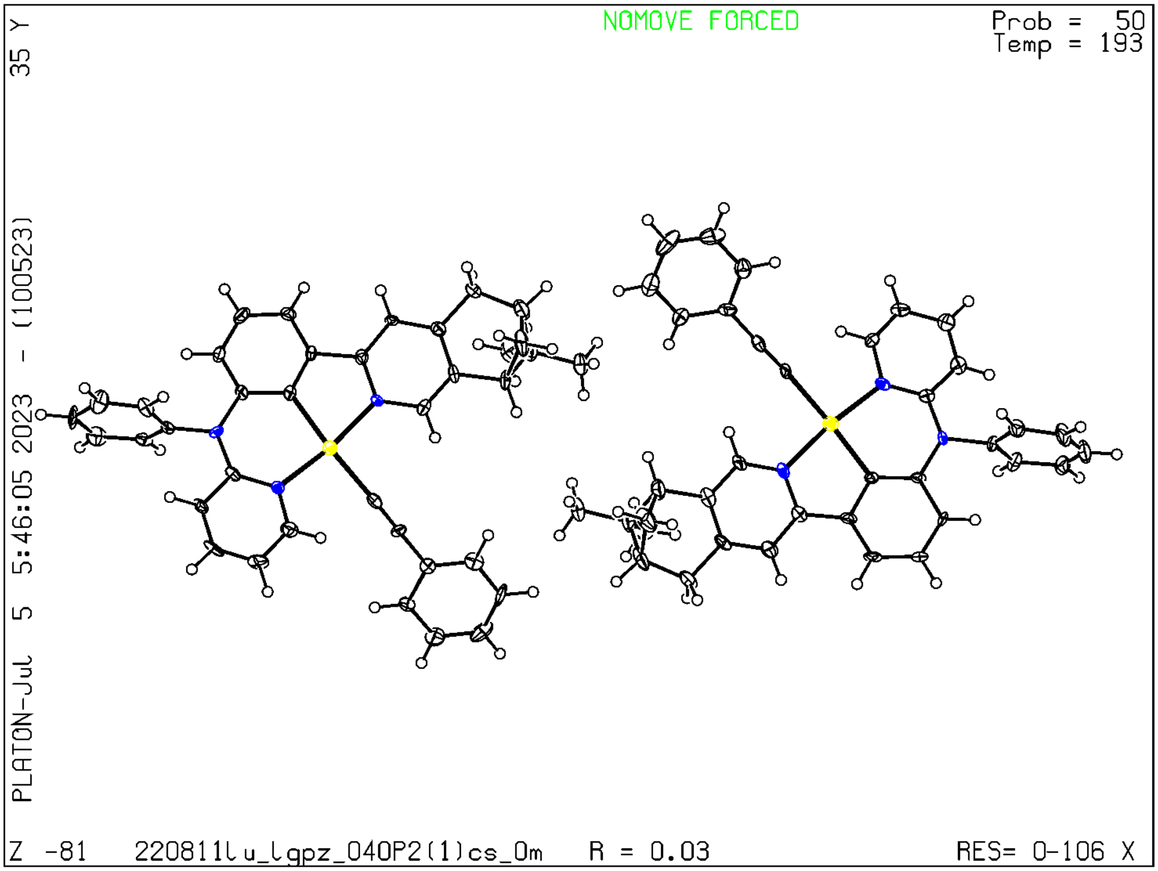

Supplement: Supplementary file 1 [file molecules-28-06369-s001.zip › checkcif-(-)-3.pdf]
