# Supplementary material for: Synthesis and Biological Activities of Luminescent 5,6-Membered Bis(Metallacyclic) Platinum(II) Complexes
Source: Molecules. 2023 Aug 31;28(17):6369. doi: 10.3390/molecules28176369 (PMC10489632; doi:10.3390/molecules28176369)
Supplement: Supplementary file 1 [file molecules-28-06369-s001.zip › Supporting Information-revised.pdf]

Synthesis and biological activities of luminescent 5,6-membered bis(metallacyclic) platinum(II) complexes

Jing Jing[1], Miao Yu[1], Lei Pan[1], Yang Zhao[1], Guo Xu[1], Hua-Hong Zhang[1], Chen Li[1], Xiao-Peng Zhang[1*]

[1] College of Chemistry and Chemical Engineering, Key Laboratory of Water Pollution Treatment & Resource Reuse of Hainan Province, Hainan Normal University, Haikou 571158, China

Email: zxp_inorganic@126.com

Characterization, crystal structures, spectroscopic properties , theoretical calculation, cytotoxicity and cell imaging

Table of Contents

1. NMR and HRMS spectra 2

2. Crystal structures 8

3. Spectroscopic properties... 10

4. Theoretical calculation.. 17

5. Cytotoxicity.. 19

6. Cell imaging.. 21

1. NMR and HRMS spectra

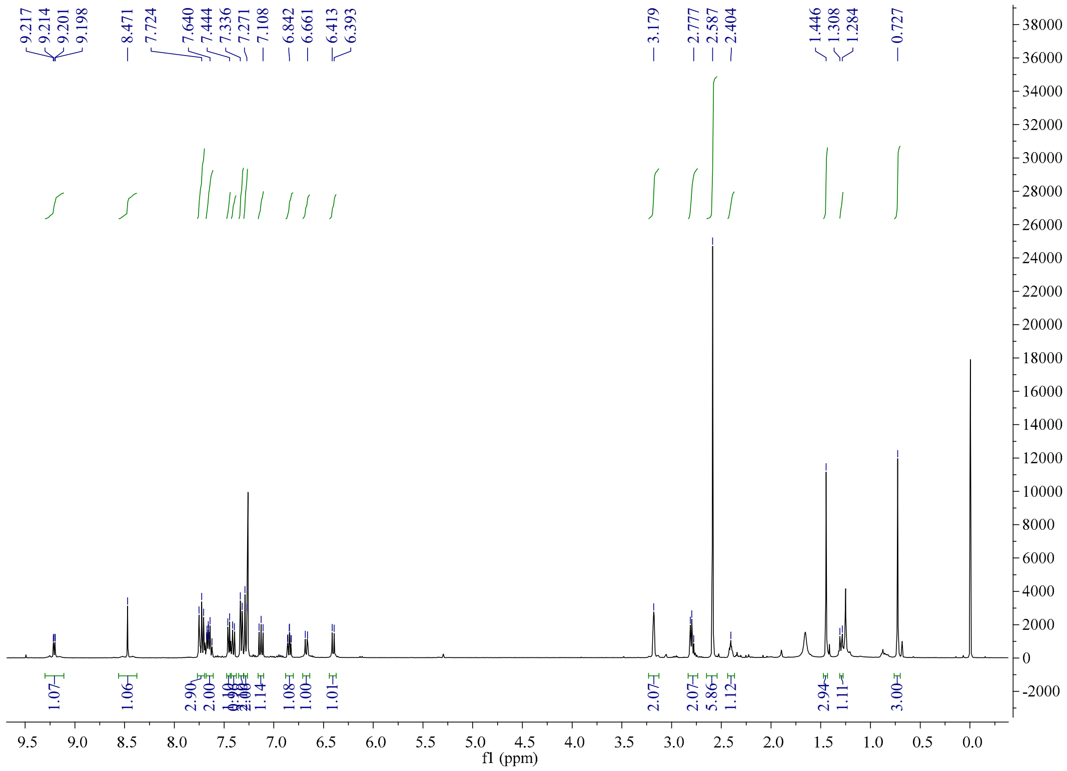

Figure S1 ^{1}H NMR of (−)-**1**

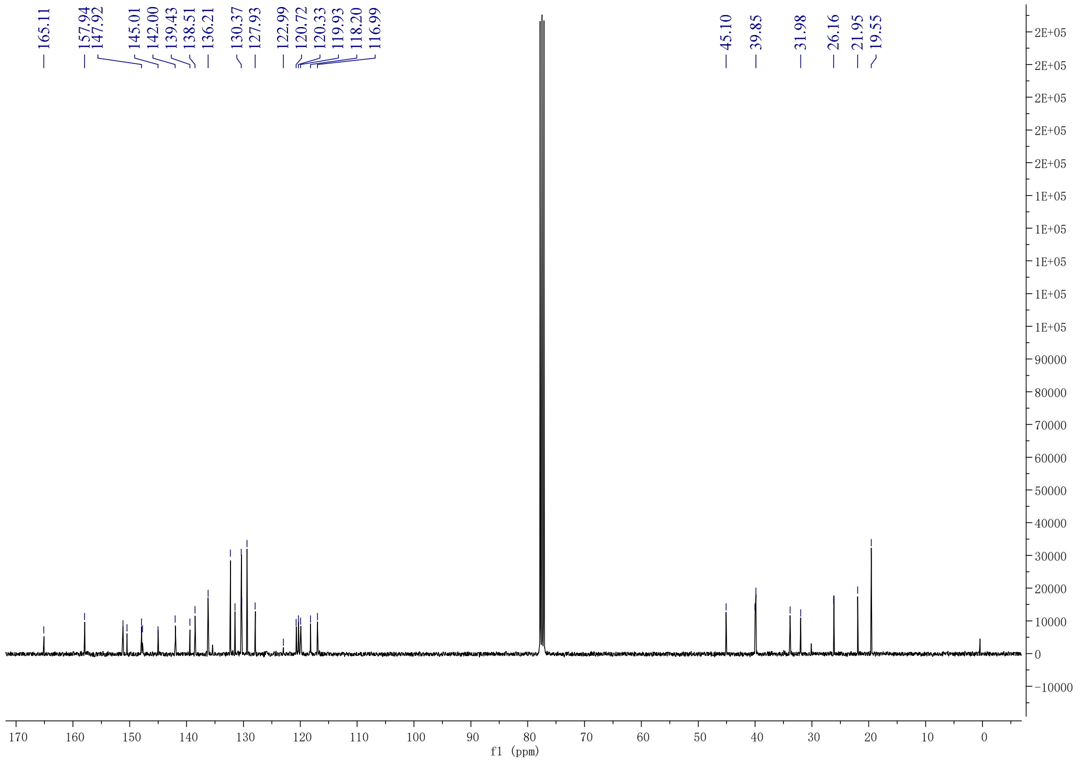

Figure S2 ^{13}C NMR of (−)-**1**

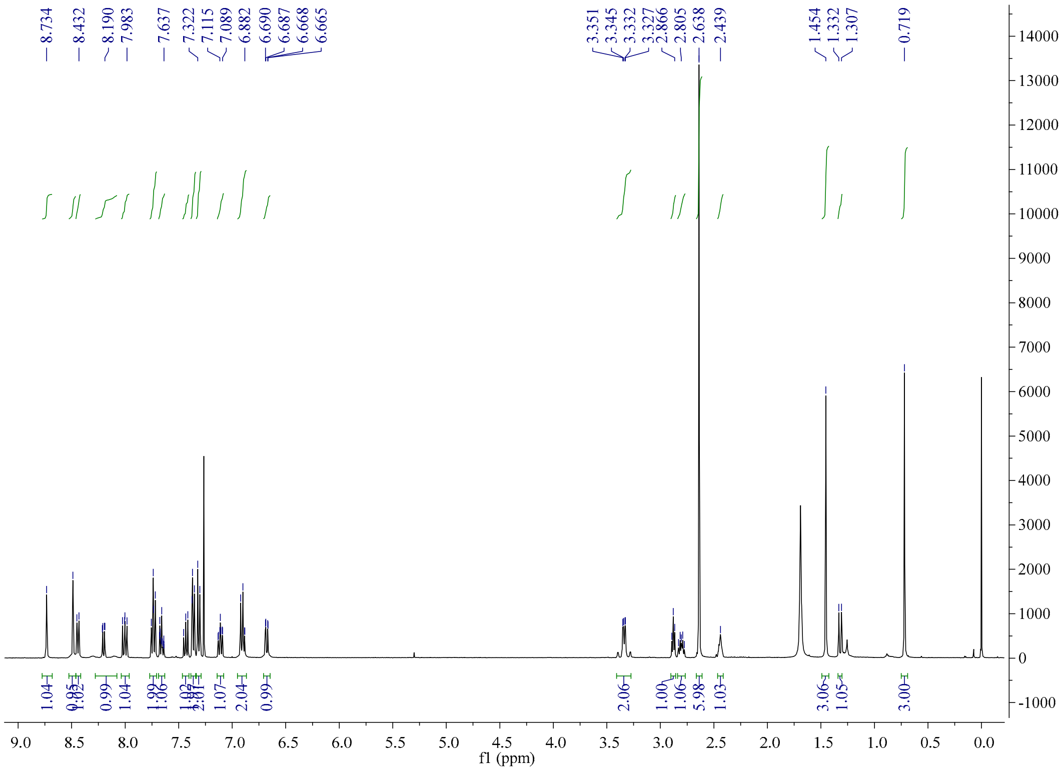

Figure S3 ^{1}H NMR of (−)-**2**

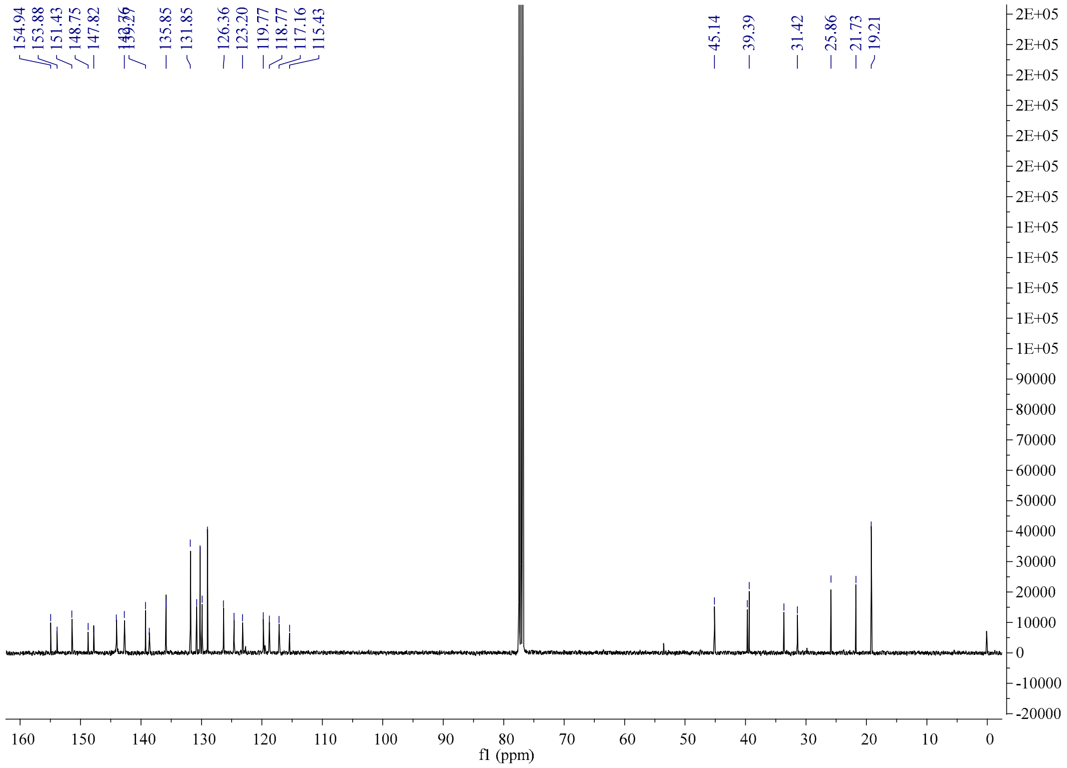

Figure S4 ^{13}C NMR of (−)-**2**

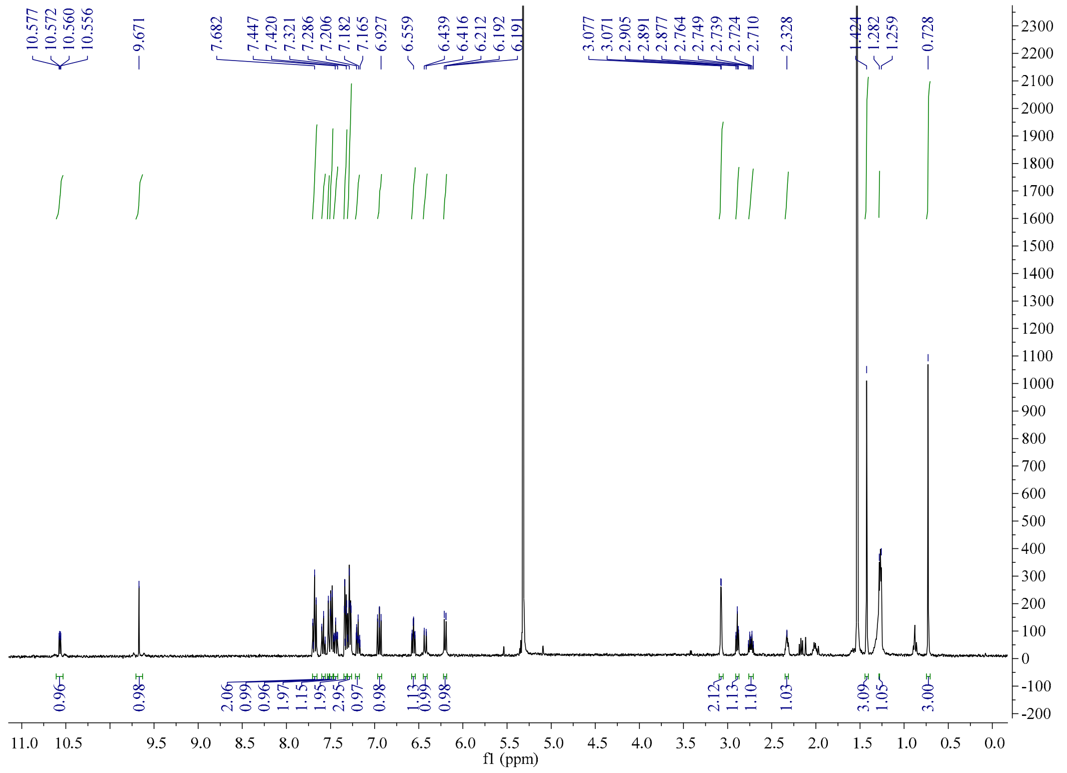

Figure S5 ^{1}H NMR of (−)-**3**

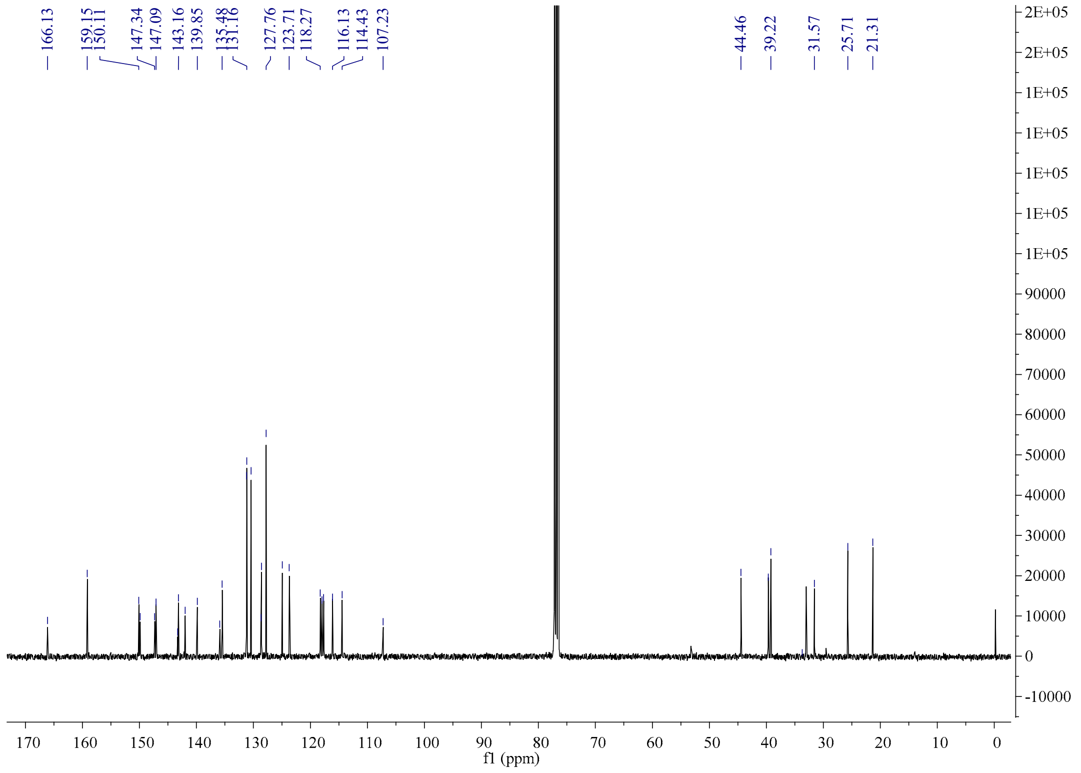

Figure S6 ^{13}C NMR of (−)-**3**

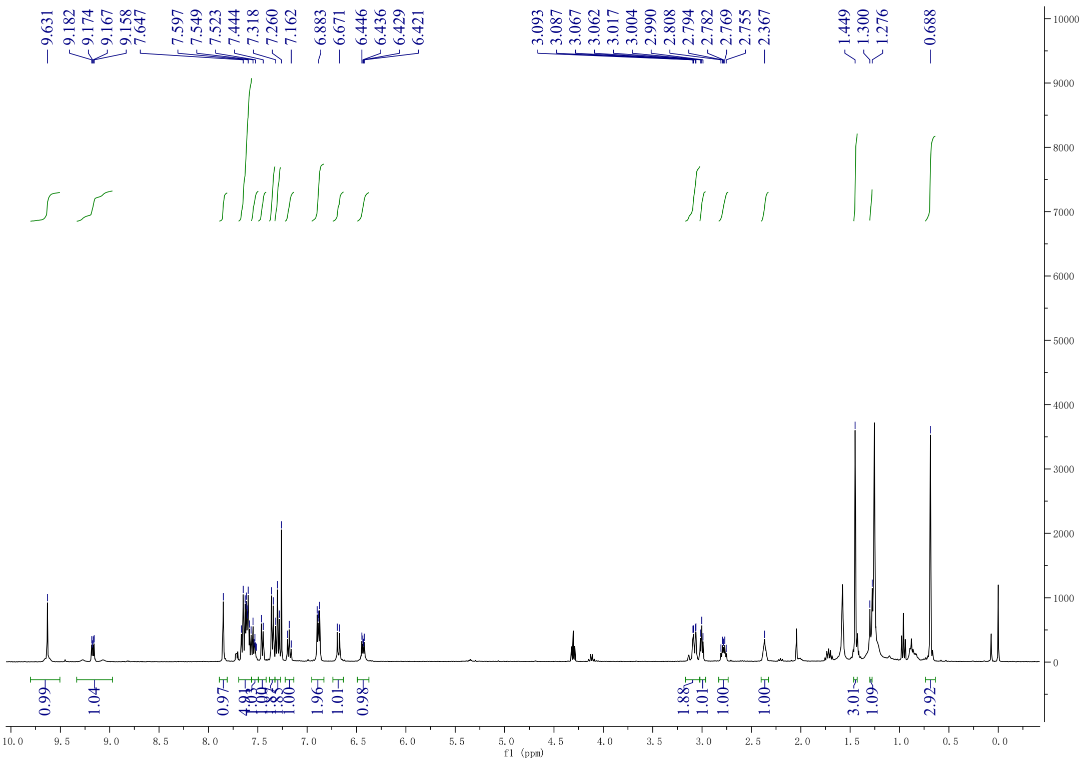

Figure S7 ^{1}H NMR of (−)-**4**

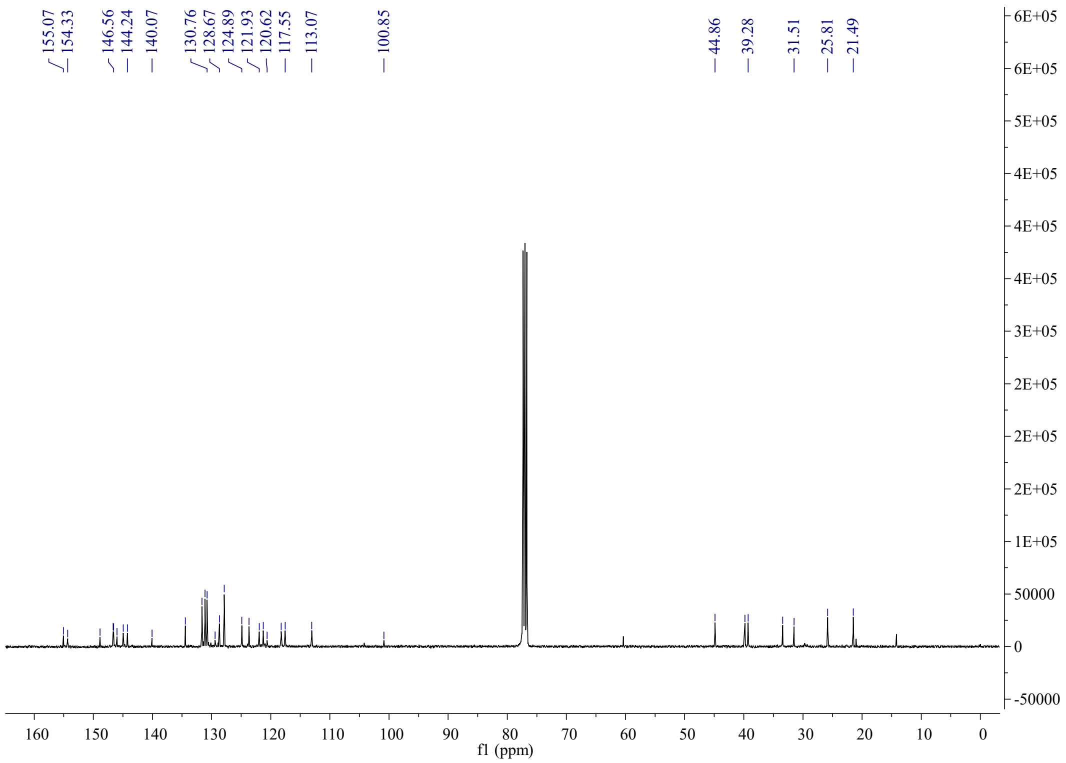

Figure S8 ^{13}C NMR of (−)-**4**

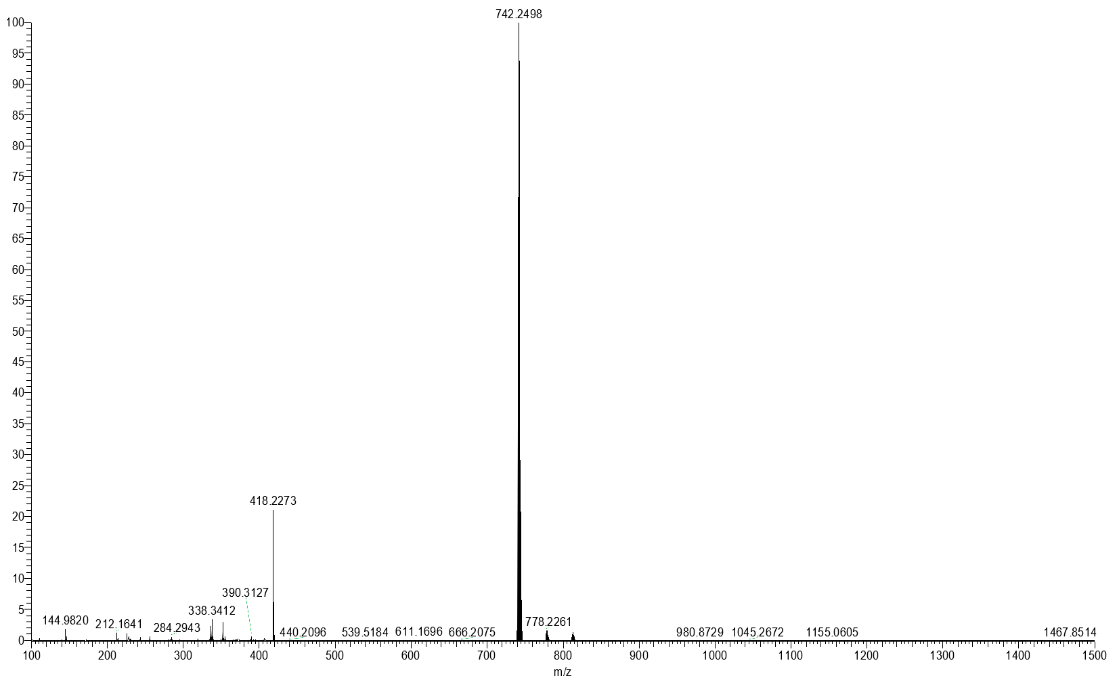

Figure S9 HRMS of (−)-**1**

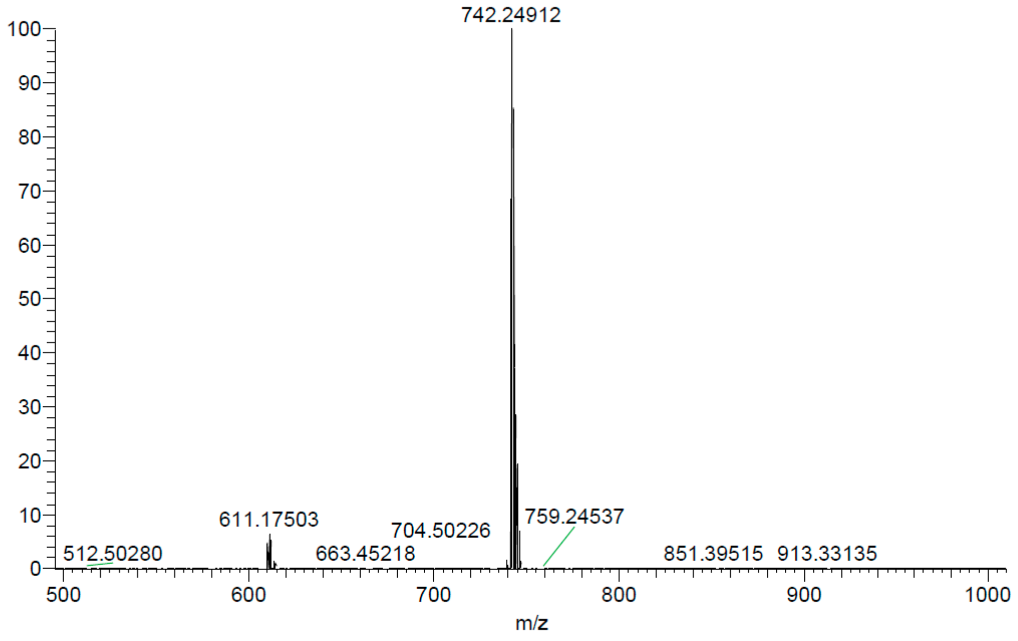

Figure S10 HRMS of (−)-**2**

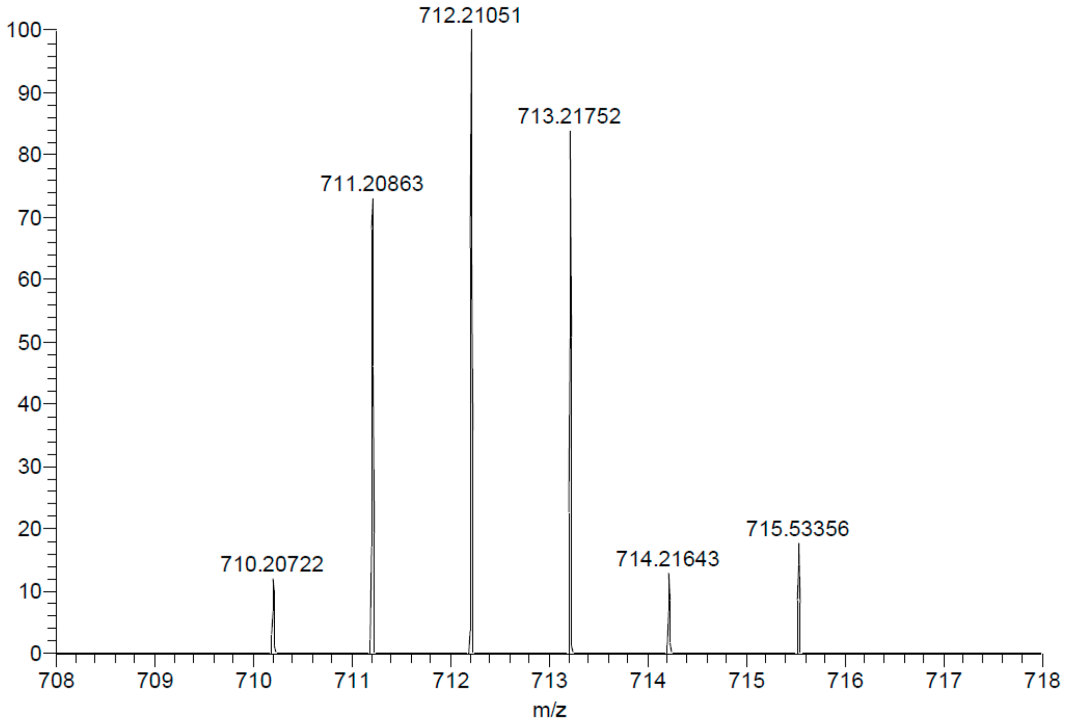

Figure S11 HRMS of (−)-3

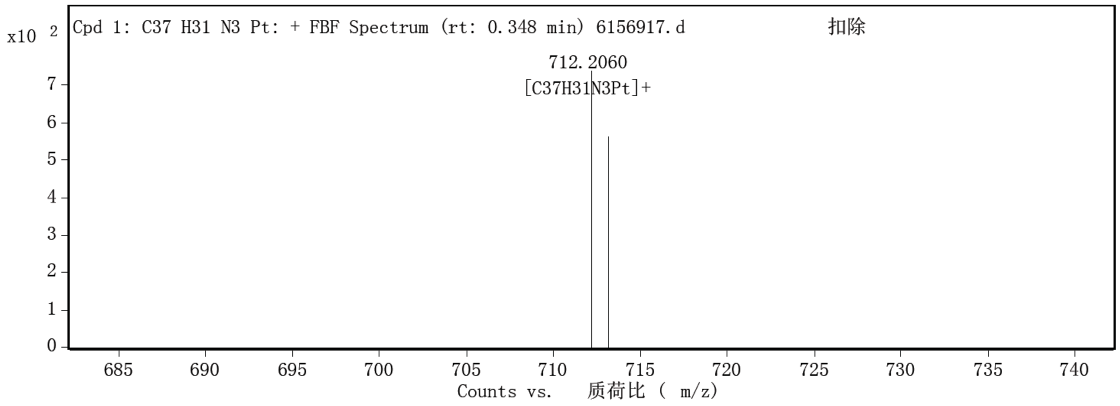

Figure S12 HRMS of (−)-4

2. Crystal structures

Table S1 Selected bond lengths (Å) of complexes (−)-**2** and (−)-**3** determined by X-ray single crystal diffraction.

Bond Lengths	(−)-**2**	(−)-**3**
Pt1−C1	2.050(8)	1.994(9)
Pt1−N1	2.072(16)	2.019(7)
Pt1−N2	2.044(19)	2.018(7)
Pt1−C2	1.89(2)	2.049(10)
Pt2−C3	2.052(8)	1.986(9)
Pt2−N3	2.086(17)	2.022(7)
Pt2−N4	2.007(19)	2.034(7)
Pt2−C4	1.97(2)	2.053(9)

Table S2 Selected bond angles () of complexes (−)-**2** and (−)-**3** determined by X-ray single crystal diffraction.

Bond Angles	(−)-**2**	(−)-**3**
C1−Pt1−N1	171.9(6)	80.3(3)
C1−Pt1−N2	92.2(6)	92.5(3)
C1−Pt1−C2	92.0(7)	168.0(4)
N1−Pt1−N2	79.8(7)	171.0(3)
N1−Pt1−C2	96.0(8)	92.8(3)
N2−Pt1−C2	175.2(9)	95.2(3)
C3−Pt2−N3	172.7(6)	82.2(3)
C3−Pt2−N4	92.4(6)	91.0(3)
C3−Pt2−C4	91.3(7)	168.7(4)
N3−Pt2−N4	81.4(7)	171.6(3)
N3−Pt2−C4	95.1(8)	91.9(3)
N4−Pt2−C4	175.5(10)	95.6(3)

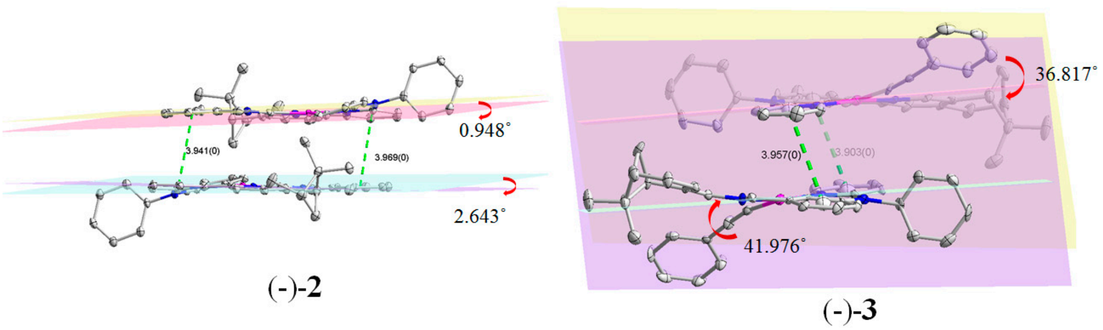

(-)-**2** (-)-**3**

Figure S13 Dihedral angles between isocyanide aryl ring or phenylacetylene and the coordination plane.

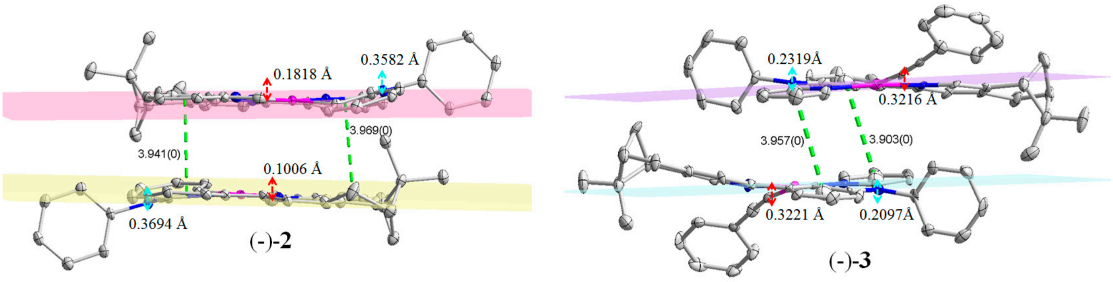

Figure S14 The distances between the C(isocyanide) or C(alkynyl) atom and the coordination plane, and the seperation between amino nitrogen atom and the coordination plane.

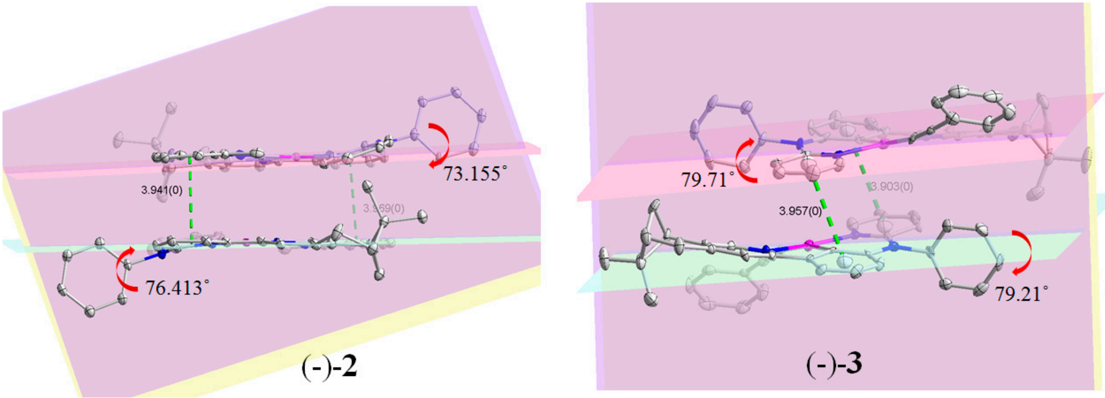

Figure S15 Dihedral angles between N-phenyl group and the coordination plane.

3. Spectroscopic properties

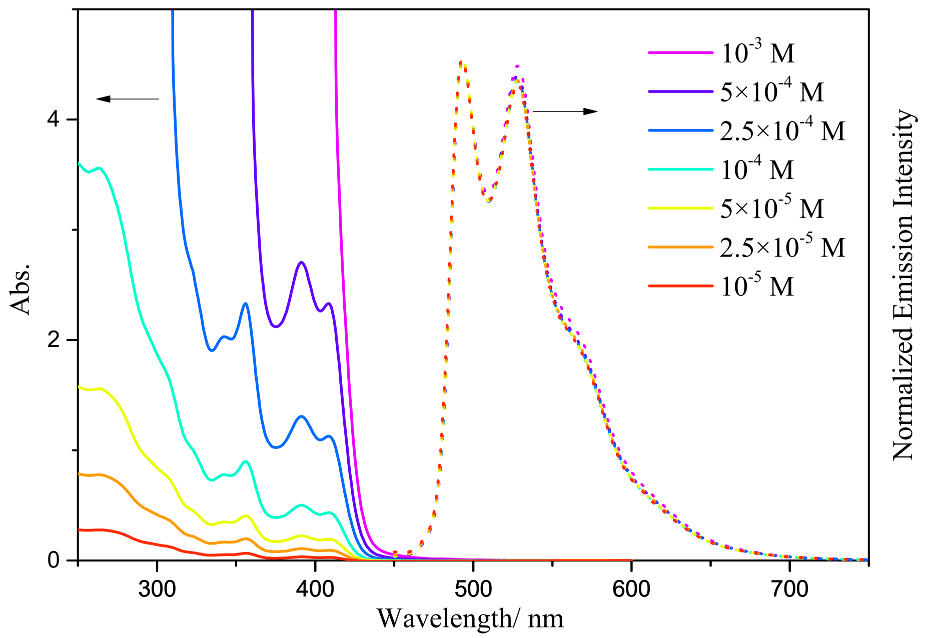

Figure S16 Absorption and emission spectra (λ_{ex} = 420 nm) of complex (−)-**1** in CH_2Cl_2 with different concentrations at RT.

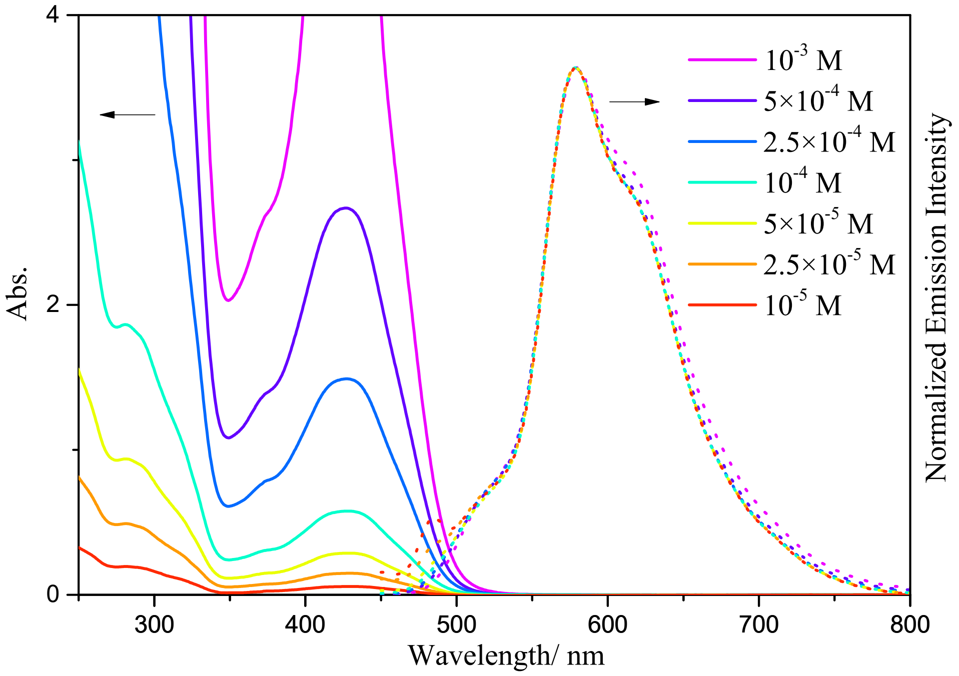

Figure S17 Absorption and emission spectra (λ_{ex} = 420 nm) of complex (−)-**2** in CH_2Cl_2 with different concentrations at RT.

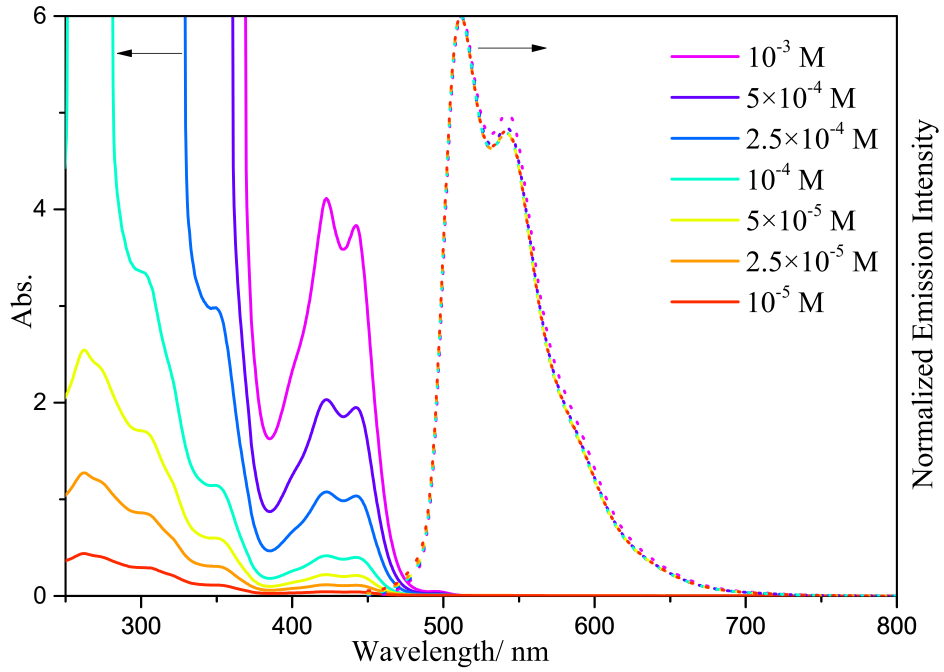

Figure S18 Absorption and emission spectra (λ_{ex} = 420 nm) of complex (−)-**3** in CH_2Cl_2 with different concentrations at RT.

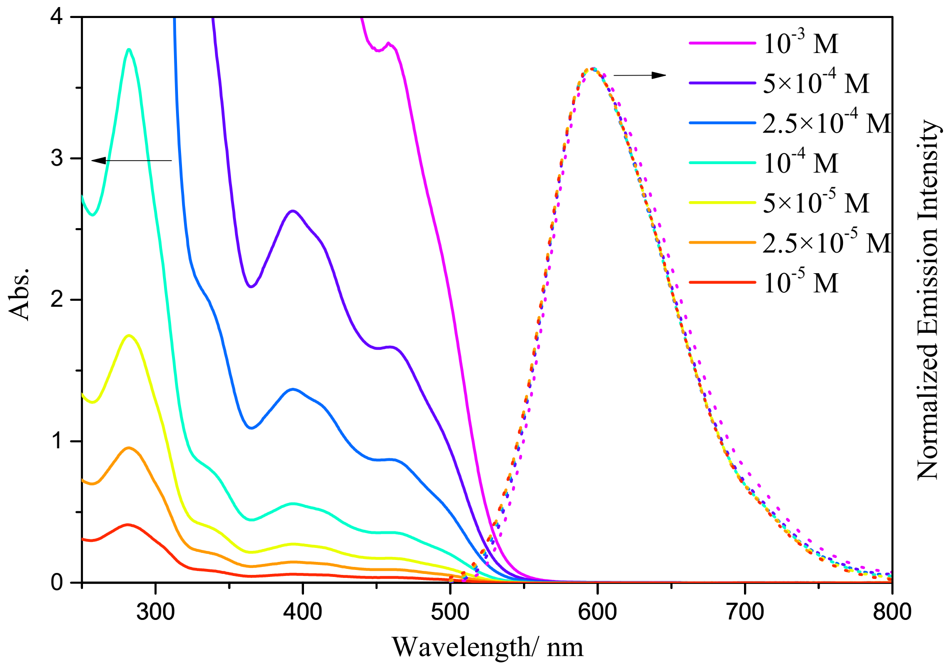

Figure S19 Absorption and emission spectra (λ_{ex} = 420 nm) of complex (−)-**4** in CH_2Cl_2 with different concentrations at RT.

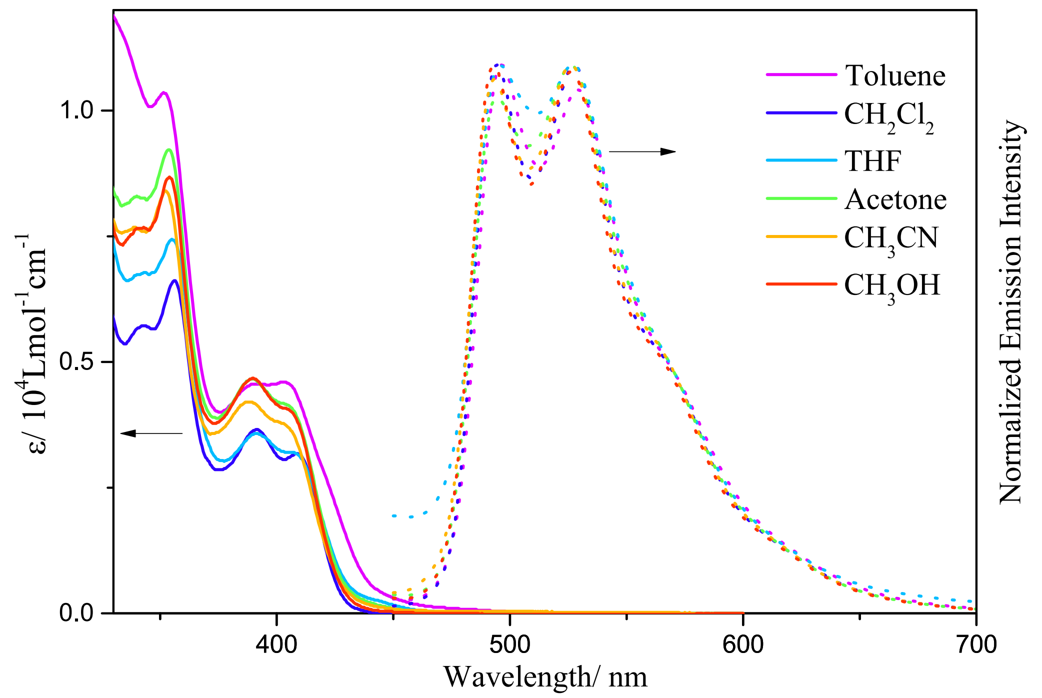

Figure S20 Absorption and emission spectra (λ_{ex} = 420 nm) of complex (−)-**1** in various solvents (5×10^{-5} mol·L^{-1}) at RT.

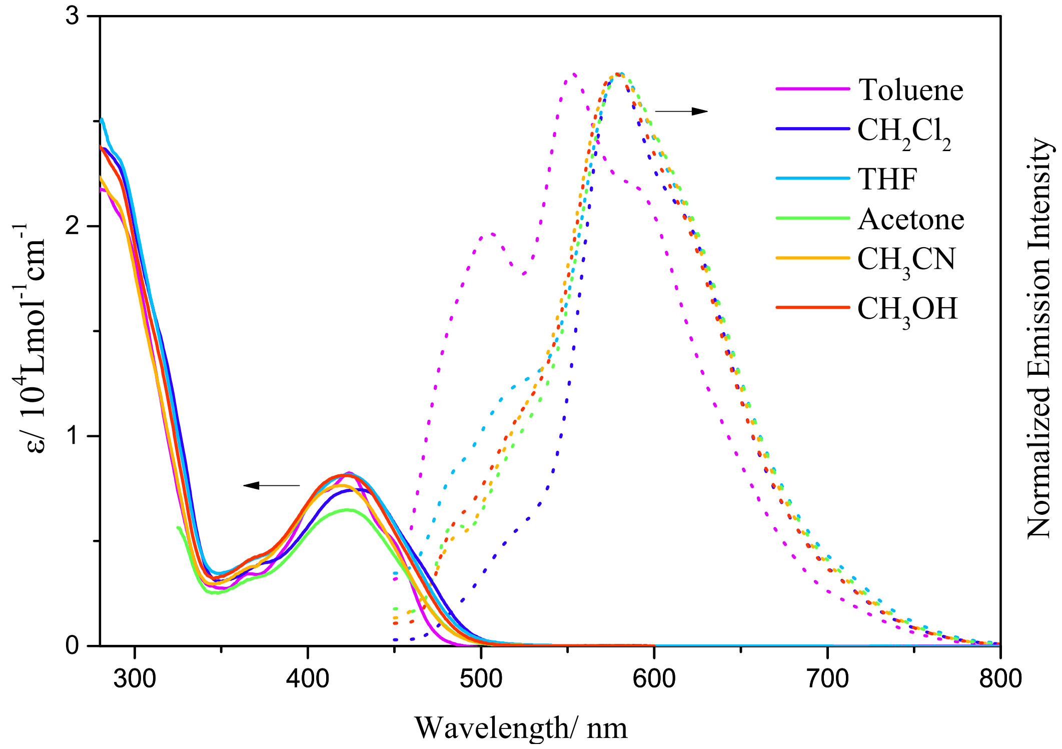

Figure S21 Absorption and emission spectra (λ_{ex} = 420 nm) of complex (−)-**2** in various solvents (5×10^{-5} mol·L^{-1}) at RT.

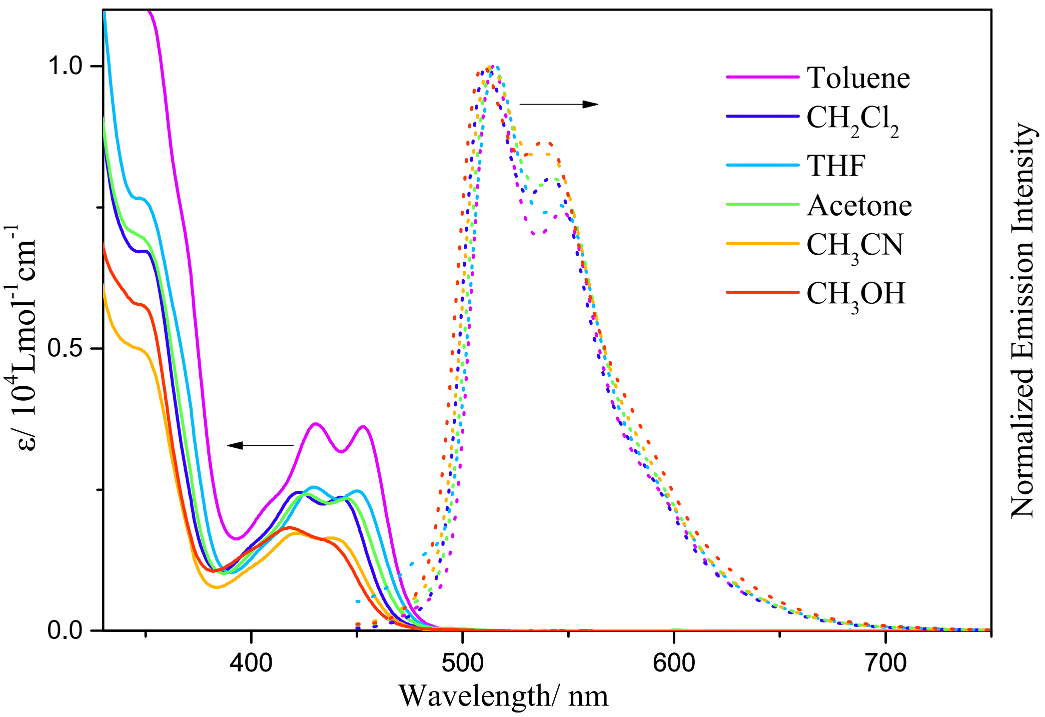

Figure S22 Absorption and emission spectra (λ_{ex} = 420 nm) of complex (−)-**3** in various solvents (5×10^{-5} mol·L^{-1}) at RT.

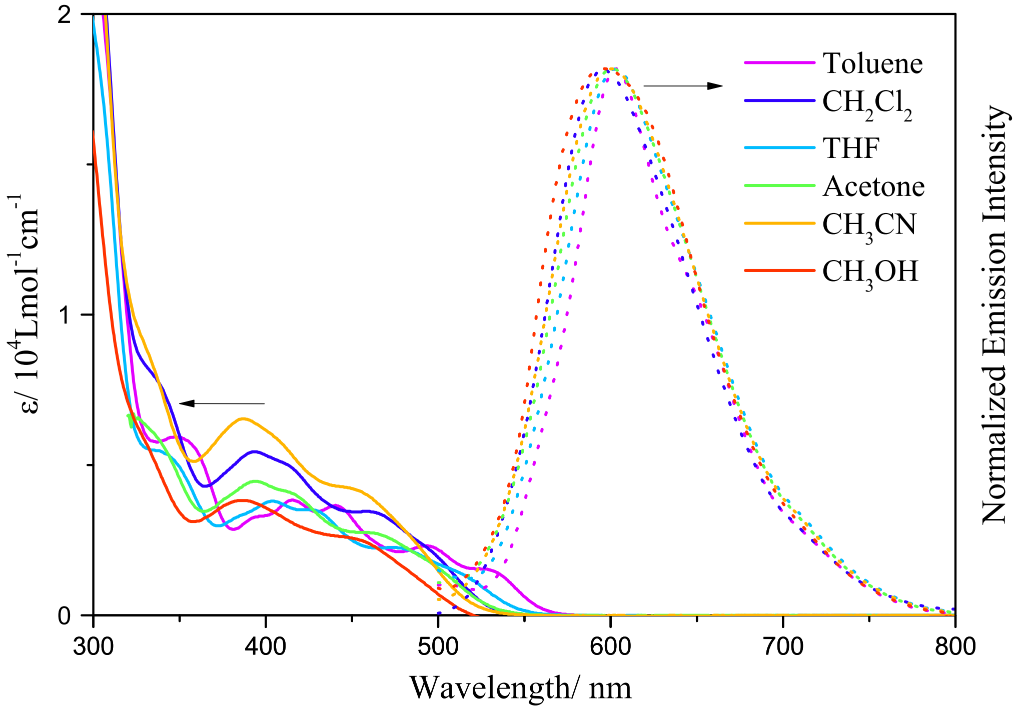

Figure S23 Absorption and emission spectra (λ_{ex} = 420 nm) of complex (−)-**4** in various solvents (5×10^{-5} mol·L^{-1}) at RT.

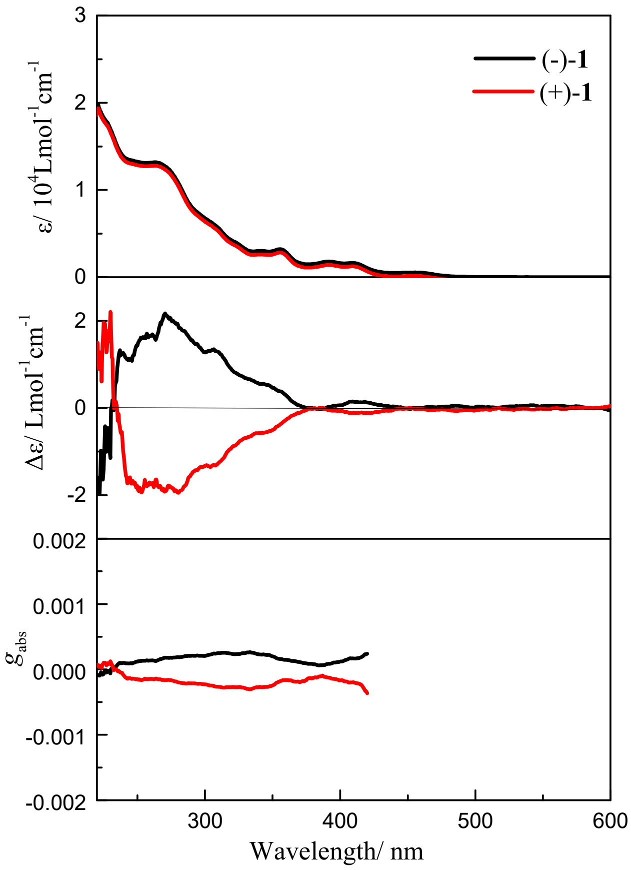

Figure S24 ECD spectra of complexes (−)-**1** and (+)-**1** in CH_2Cl_2 (5×10^{-5} mol·L^{-1}).

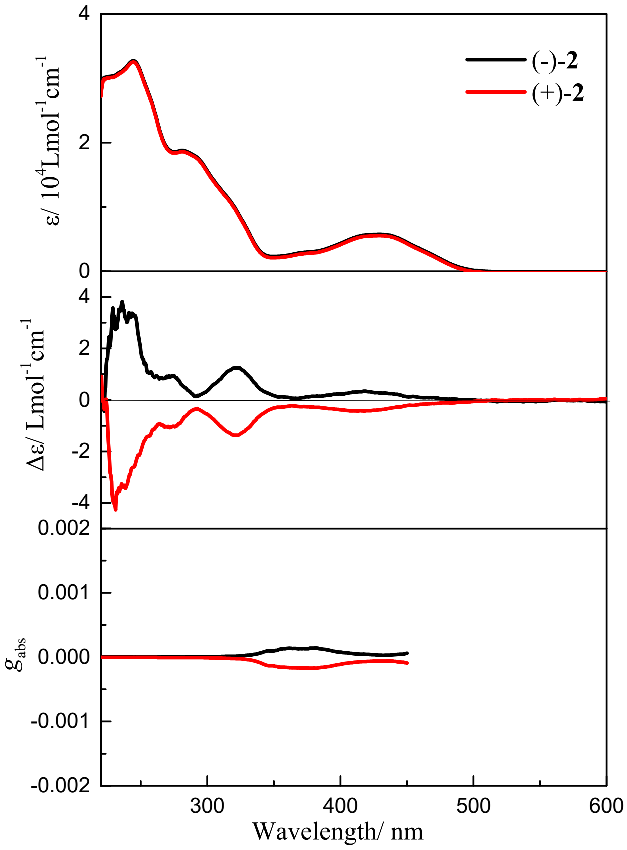

Figure S25 ECD spectra of complexes (−)-**2** and (+)-**2** in CH_2Cl_2 (5×10^{-5} mol·L^{-1}).

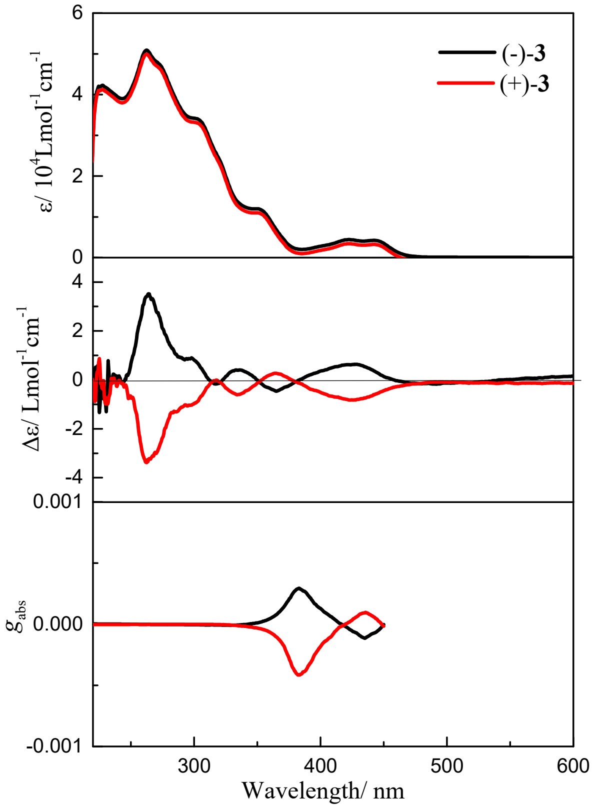

Figure S26 ECD spectra of complexes (−)-**3** and (+)-**3** in CH_2Cl_2 (5×10^{-5} mol·L^{-1}).

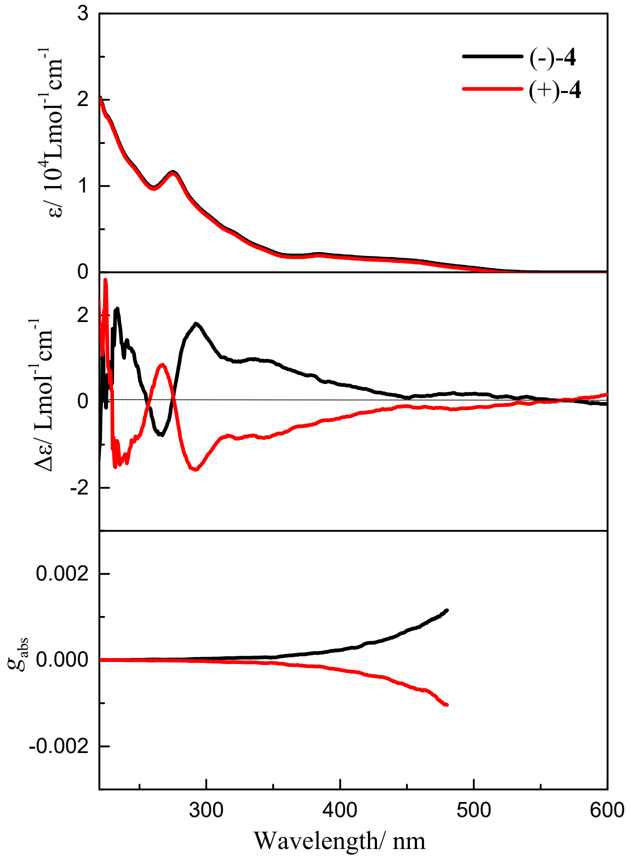

Figure S27 ECD spectra of complexes (−)-**4** and (+)-**4** in CH_2Cl_2 (5×10^{-5} mol·L^{-1}).

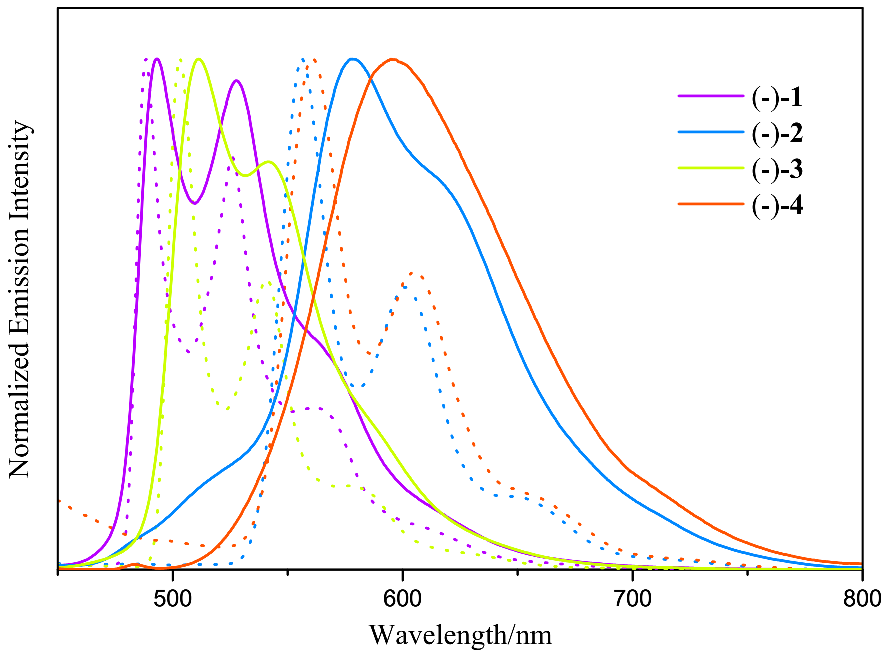

Figure S28 Emission spectra (λ_{ex} = 420 nm) of all complexes in CH_2Cl_2 (5×10^{-5} mol·L^{-1}) at RT (solid line) and in 2-methyltetrahydrofuran (5×10^{-5} mol·L^{-1}) at 77 K (dot line).

4. Theoretical calculation

Table S3 Theoretical calculation results for (–)-**1**, (–)-**2**, (–)-**3** and (–)-**4** on the basis of their optimized S_0 geometries.

Complexes	MO	Contribution percentages [%]								Main configuration of $S_0 \rightarrow S_1$ excitation/E_{cal}/λ_{cal}/f^a
(–)-**1**		Pt	Amino N	Isocyanide	**Ring A**	**Ring B**	**Ring C**	**Ring D**	**Ring E**	
	L	8.75	0.35	9.54	40.56	22.99	7.43	0.19	7.11	H→L (90.2%)
										H-1→L (7.0%)
	H	9.44	19.95	1.45	0.81	39.93	23.35	2.11	2.32	3.2497 eV
	H-1	7.49	0.96	1.09	29.33	51.18	1.64	0.74	2.57	381.53 nm
										f = 0.1271
(–)-**2**		Pt	Amino N	Isocyanide	**Ring A**	**Ring B**	**Ring C**	**Ring D**	**Ring E**	
	L	4.28	0.74	3.31	38.78	46.07	2.00	0.62	1.59	H→L (98.4%)
	H	6.67	19.08	0.73	3.45	20.36	45.38	2.25	1.32	2.9723 eV
										417.14 nm
										f = 0.1935
(–)-**3**		Pt	Amino N	Alkynyl	**Ring A**	**Ring B**	**Ring C**	**Ring D**	**Ring E**	
	L	5.82	0.29	1.19	49.98	26.39	11.33	0.15	1.44	H→L (96.6%)
	H	22.35	6.52	23.01	1.47	22.86	6.92	0.58	15.96	2.9294 eV
										423.24 nm
										f = 0.0961
(–)-**4**		Pt	Amino N	Alkynyl	**Ring A**	**Ring B**	**Ring C**	**Ring D**	**Ring E**	
	L	3.64	0.36	0.88	41.56	47.64	1.55	0.85	0.80	H→L (98.0%)
	H	20.59	7.38	25.66	0.63	6.89	19.58	0.65	18.42	2.5959 eV
										477.62 nm
										f = 0.0981

H-L represents the HOMO to LUMO transition. E_{cal}, λ_{cal} and f represent the calculated excitation energy, calculated wavelength and oscillator strength, respectively.

Table S4 NTO results for (–)-**1**, (–)-**2**, (–)-**3** and (–)-**4** on the basis of their optimized T_1 geometries.

Complexes	NTO[a]	Contribution percentages [%]							
(–)-**1**		Pt	Amino N	Isocyanide	**Ring A**	**Ring B**	**Ring C**	**Ring D**	**Ring E**
	P	4.47	0.02	1.69	46.48	42.81	1.11	0.22	0.66
	H	4.42	0.05	0.45	40.91	49.44	0.31	0.18	0.29
(–)-**2**		Pt	Amino N	Isocyanide	**Ring A**	**Ring B**	**Ring C**	**Ring D**	**Ring E**
	P	2.73	0.41	1.16	30.91	60.63	1.55	0.41	0.34
	H	1.75	16.10	0.17	12.11	44.23	22.80	1.49	0.11

(–)-**3**		Pt	Amino N	Alkynyl	**Ring A**	**Ring B**	**Ring C**	**Ring D**	**Ring E**
	P	6.74	0.04	1.63	47.31	38.44	1.59	0.35	1.43
	H	18.16	2.04	6.56	23.53	42.23	1.68	0.23	3.52
(–)-**4**		Pt	Amino N	Alkynyl	**Ring A**	**Ring B**	**Ring C**	**Ring D**	**Ring E**
	P	3.85	0.43	0.93	33.61	56.21	1.80	0.53	0.69
	H	11.07	16.60	5.00	5.15	26.14	31.31	1.30	2.87

[a] H and P represent NTO hole and particle orbitals, respectively.

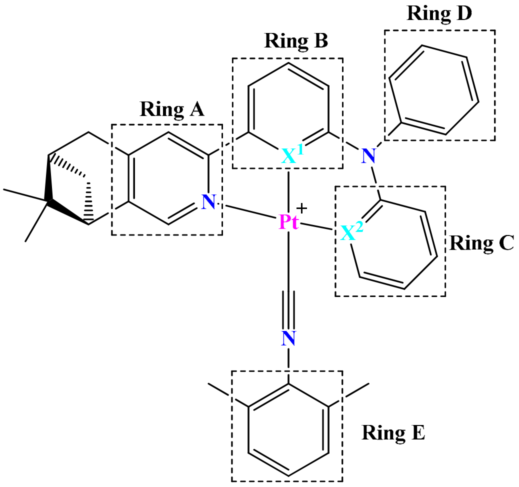

(-)-**1**, X^1 = C, X^2 = N

(-)-**2**, X^1 = N, X^2 =C

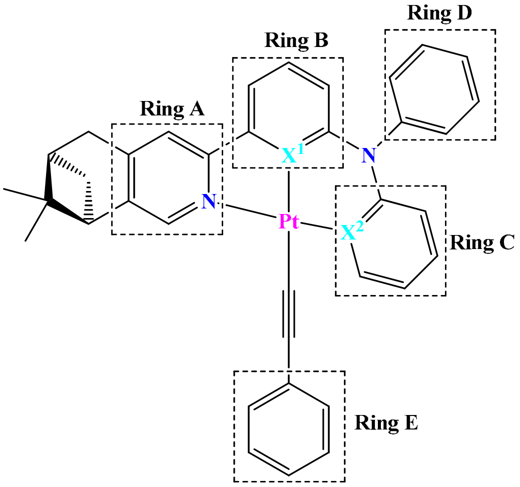

(-)-**3**, X^1 = C, X^2 = N

(-)-**4**, X^1 = N, X^2 =C

5. Cytotoxicity

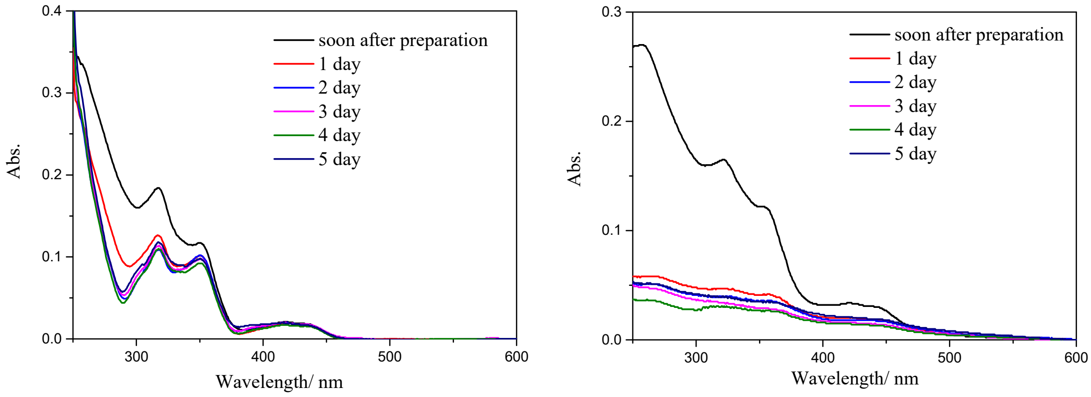

Figure 29 The temporal change of absorption spectra of Pt(N^C*N')Cl in DMSO (left) and DMSO-PBS (v/v = 5:95) (right) by heating at 37 °C for 5 days (10.0 μM).

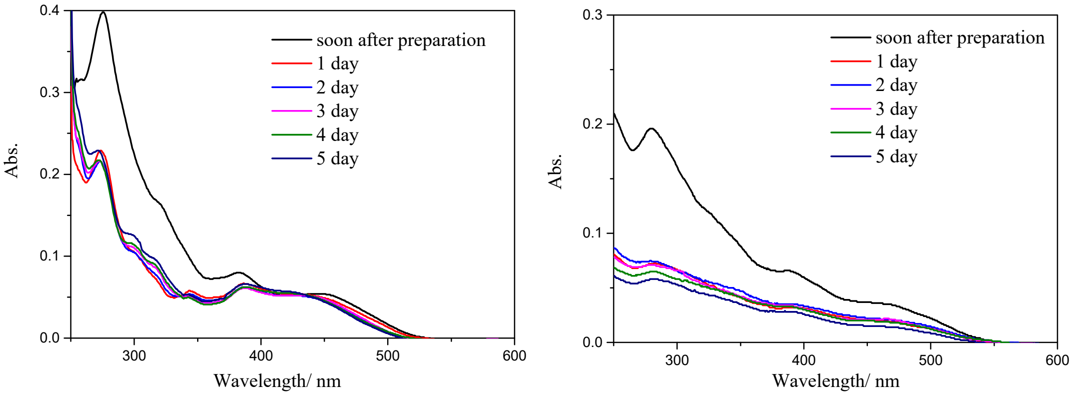

Figure 30 The temporal change of absorption spectra of Pt(N^N'*C)Cl in DMSO (left) and DMSO-PBS (v/v = 5:95) (right) by heating at 37 °C for 5 days (10.0 μM).

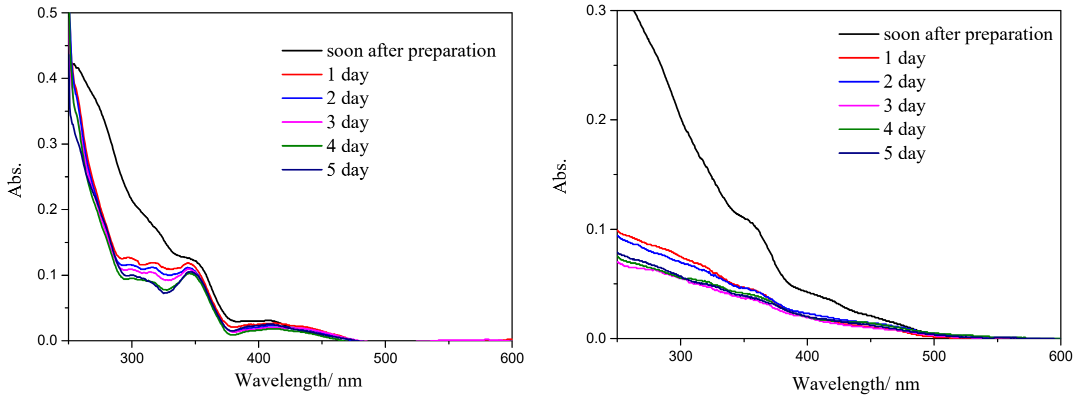

Figure 31 The temporal change of absorption spectra of (-)-**1** in DMSO (left) and DMSO-PBS (v/v = 5:95) (right) by heating at 37 °C for 5 days (10.0 μM).

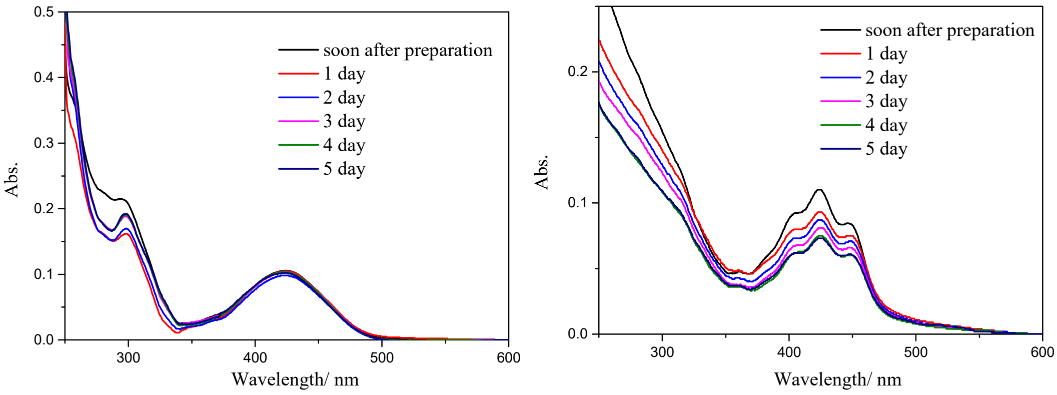

Figure 32 The temporal change of absorption spectra of (-)-**2** in DMSO (left) and DMSO-PBS (v/v = 5:95) (right) by heating at 37 °C for 5 days (10.0 μM).

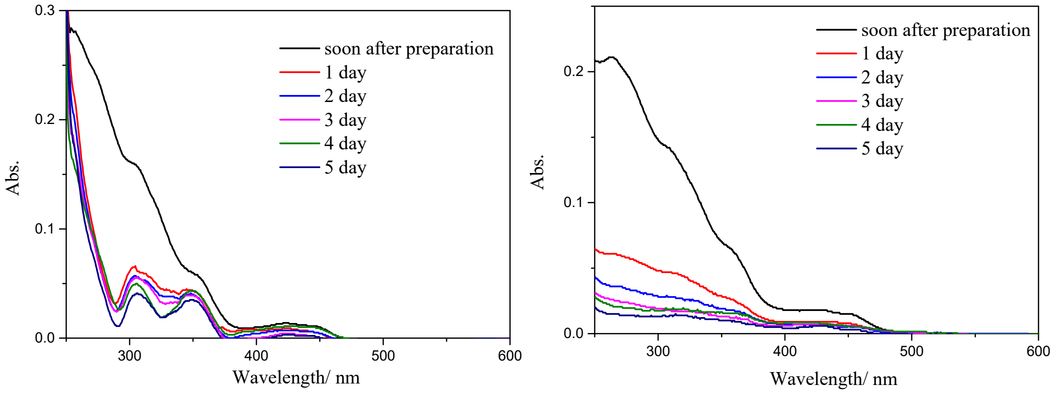

Figure 33 The temporal change of absorption spectra of (-)-**3** in DMSO (left) and DMSO-PBS (v/v = 5:95) (right) by heating at 37 °C for 5 days (10.0 μM).

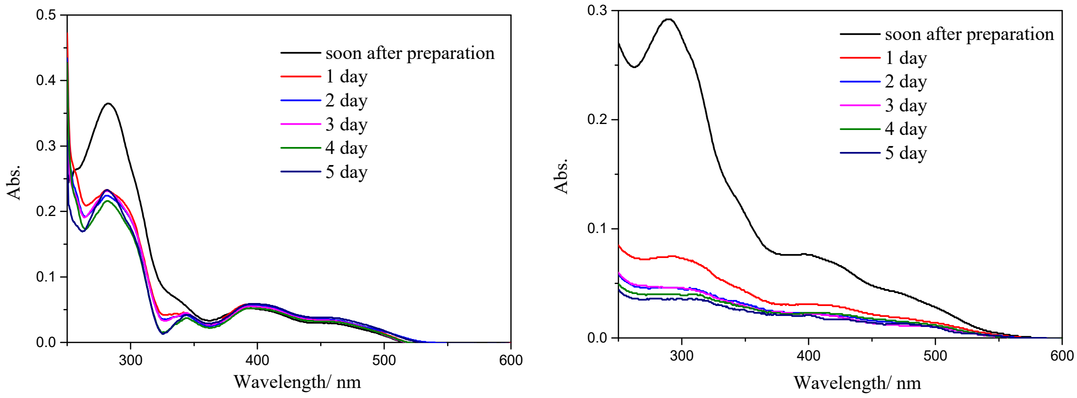

Figure 34 The temporal change of absorption spectra of (-)-**4** in DMSO (left) and DMSO-PBS (v/v = 5:95) (right) by heating at 37 °C for 5 days (10.0 μM).

6. Cell imaging

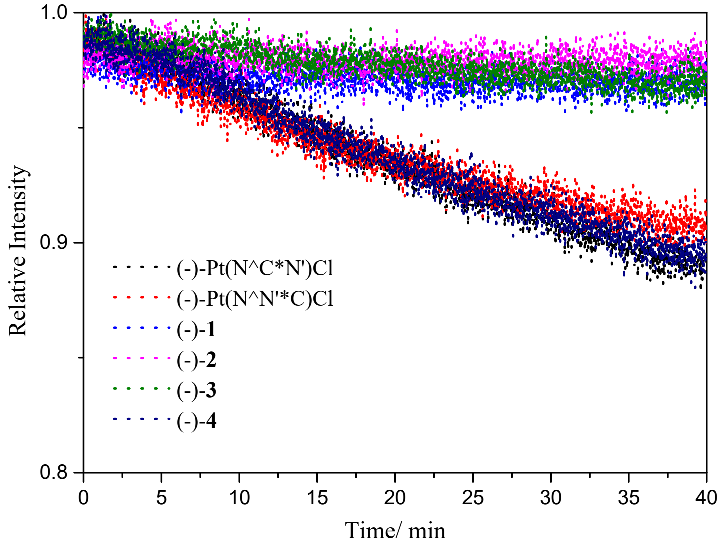

Figure S35 The photostability figures of all complexes (10.0 μM). The luminescence intensity under continuous excitation at 420 nm for 40 min.
